# QT interval is correlated with and can predict the comorbidity of depression and anxiety: A cross-sectional study on outpatients with first-episode depression

**DOI:** 10.3389/fcvm.2022.915539

**Published:** 2022-09-29

**Authors:** Mingcong Tang, Juzhe Xi, Xiwang Fan

**Affiliations:** ^1^Shanghai Key Laboratory of Mental Health and Psychological Crisis Intervention, Affiliated Mental Health Center (ECNU), School of Psychology and Cognitive Science, East China Normal University, Shanghai, China; ^2^Department of Psychology, Southwest University, Chongqing, China; ^3^Clinical Research Center for Mental Disorders, Shanghai Pudong New Area Mental Health Center, School of Medicine, Tongji University, Shanghai, China

**Keywords:** depression disorders, anxiety disorders, psychiatric symptoms, ECG abnormalities, cardiovascular disorders

## Abstract

**Object:**

Patients with depression are at an increased risk for developing cardiovascular diseases. The associations between electrocardiogram (ECG) abnormalities and the severity of psychiatric disorders, such as depression and anxiety, have not been clearly elucidated. The present study aims to investigate the associations between depression and anxiety symptoms with ECG indices, and to predict the severity of depression and anxiety using ECG indicators.

**Methods:**

61 outpatients with first-episode depression from the Shanghai Pudong New Area Mental Health Center were selected and met the diagnostic criteria of DSM-IV. All participants provided self-reported scores on the Zung Self-Rating Depression Scale (SDS) and Zung Self-Rating Anxiety Scale (SAS) and underwent the standard 12-lead ECG assessment.

**Results:**

Among the 61 included outpatients (mean [standard deviation, SD] age: 37.84 [13.82] years; 41[67.2%] were female), there were 2 (3.3%) outpatients without depression symptoms, 16 (26.2%) with mild depression, 19 (31.1%) with moderate depression, and 24 (39.3%) with severe depression. Ten (16.4%) outpatients did not have anxiety symptoms, 19 (31.1%) exhibited mild anxiety, 20 (32.8%) exhibited moderate anxiety, and 12 (19.7%) exhibited severe anxiety. Only 1 (1.6%) outpatient exhibited neither depression nor anxiety, 9 (14.8%) and 1 (1.6%) outpatients only exhibited depression and anxiety, respectively, and most outpatients (50 [82.0%]) had comorbid depression and anxiety symptoms. In the correlation analysis, depression and anxiety severity levels were significantly positively correlated (r = 0.717, *p* < 0.01). Moreover, categorical anxiety significantly differs in QT interval (*p* = 0.022), and continuous SAS scores were significantly correlated with QT interval (r = 0.263, *p* = 0.04). In addition, the correlations between ECG measurements and both categorical depression and continuous SDS scores were not statistically significant. The comorbidity of anxiety and depression was significantly correlated with heart rate (*p* = 0.039) and QT interval (*p* = 0.002). Disorder status significantly differed with different QT intervals (*p* = 0.021). In the prediction analysis, QT interval was the only significant predictor (*p* = 0.01, b = 0.058, Odds Ratio = 1.059) for comorbid anxiety and depression symptoms.

**Conclusion:**

This study found that comorbid symptoms of depression and anxiety were significantly associated with QT interval and heart rate. Additionally, QT interval could predict the comorbidity of these two psychiatric disorders. Further prospective research in a larger and high-risk population is needed.

## Introduction

Psychiatric disorders, including depression and anxiety, are common in patients with coronary artery diseases, which are a major cause of death around the world (1). Previous research found that depression and heart disease are very common and often coexist (2). Similarly, anxiety is associated with an increased risk of cardiovascular diseases, especially heart failure (3–5). One explanation for the widely observed associations between cardiovascular diseases and depression and anxiety is that depression and anxiety lead to altered inflammation status and sympathetic nervous system activity that may adversely affect the cardiovascular system (2, 6).

Electrocardiogram (ECG) is a non-invasive measure of cardiovascular functions that can reflect the balance between the sympathetic and parasympathetic divisions of the autonomic nervous system. Researchers have found significant correlations between ECG indices and psychological disorders (7). For example, prolongation of QT interval has been related to depression symptoms in female patients with acute coronary syndrome (8). In addition, anxious participants were found to have a significantly lower respiratory sinus arrhythmia (RSA) compared with healthy controls (9). Since there has been strong comorbidity between anxiety and depression (10–12), both of them were found to be related to decreased heart rate variability (HRV) (13–15). However, the impact of depression and anxiety symptoms on ECG measurements may depend on the type of anxiety (e.g., general anxiety or heart-focused anxiety) and the stage of depression (e.g., onset, maintenance or recurrent) (16). Additionally, researchers developed a model based on recurrent neural network (RNN) (a deep learning-based model) and long short-term memory (LSTM) autoencoder to predict the risk of depression based on ECG measurements (17). This model could differentiate between “normal,” “abnormal,” and “risky” heartbeats, which correspond to different severity levels of depression. Although many studies have evaluated the associations of psychiatric disorders with ECG measurements, they only focused on a few ECG indices.

Given that mental impairments can influence physiological functions, we assume that physiological presentations may in turn reflect mental health conditions. Moreover, compared with other depression and anxiety severity physiological evaluation methods (e.g., EEG, EMG, saliva tests, and Dermal electricity), the use of ECG is much more convenient and accessible, and a link between ECG to anxiety and depression symptoms could help us form a straightforward understanding of the psychosomatic disease. To our knowledge, how ECG measurements predict psychiatric disorders has been less studied previously. Therefore, we tried to fill this knowledge gap by predicting the severity of depression and anxiety symptoms based on main ECG indices.

In this cross-sectional study, we conducted correlation analyses of psychological and physiological data to investigate the association of depression and anxiety symptoms with ECG indices (namely heart rate, PR interval, QT interval, corrected QT interval [QTC], and QRS complex). Then we performed prediction analyses to evaluate whether the extracted ECG indices can predict symptom severity of depression and anxiety.

## Materials and methods

The present study involving human participants was reviewed and approved by the Shanghai Pudong New Area Mental Health Ethics Committee. The outpatients/participants provided their written informed consent to participate in this study.

### Recruitment of participants

In this study, 61 outpatients were originally recruited from the Shanghai Pudong New Area Mental Health Center. The following were the inclusion criteria: (1) met the diagnostic criteria for depression in the Diagnostic and Statistical Manual of Mental Disorders-IV (DSM-IV); (2) aged between 18 and 70; (3) were male or female; (4) had no history of head trauma, obvious intellectual disability, or other serious or uncontrolled stable physical illness. The outpatients with asthma or respiratory allergy, sensitivity to plant extracts, olfactory problems, nasal injury, other comorbid psychological diseases, previous cardiovascular diseases, and history of myocardial infarction, those who had undergone coronary artery bypass surgery and percutaneous transluminal coronary angioplasty, those who had previously experienced arrhythmia (atrial fibrillation, ventricular atrial block, left and right heart block, etc.), had chronic bronchitis, atelectasis, and other chronic respiratory diseases, and those who had or were receiving other psychotropic-relevant medications (sodium channel blockers, QTc-prolonging drugs, first-generation antipsychotics, second-generation antipsychotics, antidepressants, cardiovascular drugs (nitrates, β-blockers, calcium channel antagonists, antithrombotics), and lipid-lowering drugs, etc.) before the ECG recordings were excluded. Enrollment and assessment were conducted from January 2021 to September 2021. Data were collected from October 2021 to January 2022 for analysis.

The G^*^ Power (Version 3.1) – *Post Hoc* power analysis (two-tails) revealed that the present sample size (*n* = 61) was sufficient for the detection of a correlation coefficient of r = 0.40 and a moderate effect size (0.25) difference among individuals with different severity levels of psychological disorders, with a power (1-β) level of 0.90 and a significant α level of 0.05.

Demographic and clinical information, including age, sex, education level, self-reported depression and anxiety scores, and five ECG indices (heart rate, PR interval, QT interval, QTC, and QRS complex), was collected.

### Instruments and assessment

#### Assessment of the severity of depression

The severity of depression was assessed using the Zung Self-Rating Depression Scale (SDS) (18). The SDS consists of 20 items with a 4-point Likert-type scale for each item. Therefore, the total raw score of this scale ranges from 20 to 80. The total raw score will then be multiplied 1.25 times to give rise to an SDS score. Based on the SDS score, subjects are classified as normal (<53), having mild depression (53 to 62), having moderate to major depression (63 to 72), and having severe to extreme major depression (>72).

#### Assessment of the severity of anxiety

The Zung Self-Rating Anxiety Scale (SAS) was utilized to assess participants' anxiety severity (19). The SAS includes 20 items covering a variety of anxiety symptoms, both psychological (e.g., “I feel afraid for no reason at all” and “I feel like I'm falling apart and going to pieces”) and somatic (e.g., “My arms and legs shake and tremble” and “I feel my heart is beating fast”). Responses are given on a 4-point scale. Participants are instructed to answer the questions based on their experiences (either negative or positive) over the last week, with positive experiences being reversely scored from 4 to 1. The total raw score for SAS ranges from 20 to 80 and will be multiplied 1.25 times to yield an SAS score. Based on the SAS score, subjects are classified as normal (<50), experiencing mild anxiety (50 to 59), experiencing moderate to major anxiety (60 to 69), and experiencing severe to extreme major anxiety (>69).

#### ECG measurements

Participants were subject to the standard 12-lead and a 5-min ECG to assess cardiac functions. ECGs were recorded using a standard 12-lead tracing at rest in the supine position with a speed of 25 mm/s, an amplitude of 10 mm/mV, and a sampling frequency of at least 500 Hz. We collected the PR interval (ms), QRS interval (ms), and QT interval (ms) as ECG data. The PR interval was the time from the onset of the P wave to the start of the QRS complex. This is an indication of atrioventricular node conduction. The QT interval was the time from the start point of the QRS complex, expressed as ventricular depolarization, to the return point (visualized) of the T wave. It results from ventricular repolarization. The corrected QT (QTc) interval was obtained by the tangent method and corrected for heart rate using Bazett's formula: QTc = QT/√RR (20). This formula has been commonly used and well established in previous research (21–23). Automated analysis was performed through a digitized multi-channel computer-assisted program (GE 12SL ECG Analysis), which uses validated algorithms for ECG parameters measurement. ECG analysis is described elsewhere (24).

#### Statistical analysis

Software packages Statistical Product and Service Solutions (SPSS, Version 26.0) and R (Version 4.0.3) were used for all statistical analyses. Prior to analysis, variables were screened for accuracy of data entry, missing values, outliers and compliance with the assumptions of univariate analysis, such as normality test. Missing values were imputed with the median for continuous variables. For descriptive analysis, clinical and demographic data are presented as mean ± standard deviation or mean ± standard error of the mean for continuous variables, and number (percentage) for categorical variables. Two-tailed *P* < 0.05 was considered statistically significant.

##### Coding for the participants' basic information

The gender of the participants was coded as follows: “1” for male and “2” for female. The education level of the participants was coded as follows: “1” for primary school-educated; “2” for junior high school or equivalent-educated; “3” for high school or equivalent-educated; “4” for junior college-educated; “5” for undergraduate-educated; and “6” for postgraduate-educated. Categorical values of depression and anxiety were determined based on the symptom severity according to the classification criteria for continuous SDS and SAS scores mentioned above as follows: “1” for normal; “2” for mild symptom; “3” for moderate symptom; and “4” for severe symptom. The disorder status (anxiety only, depression only, comorbid anxiety and depression, and no symptom/low severity of both anxiety and depression) was coded based on comorbidity: 1 for anxiety-only, 2 for depression-only, 3 for comorbid anxiety and depression, and 4 for no symptom/low severity of both.

##### Determination of the associations of demographic indicators with depression, anxiety, and ECG measurements

As for the correlation between demographic indicators and ECG measures, Pearson product-moment correlation was conducted for the relationship between age and ECG measures. Independent sample *T*-test was used to assess the association between participants' sex and ECG measures, and One-way ANOVA was performed to evaluate the relationship between education levels and ECG measures.

In terms of demographic indicators and symptom scores, Pearson correlation was conducted for the relationship between age and continuous SDS and SAS scores. Independent sample *T*-test was used to assess the association between participants' sex and continue SDS and SAS scores. One-way ANOVA was performed to evaluate the relationship between education levels and continue SDS and SAS scores, and age and categorical SDS and SAS values. Moreover, a chi-square test was carried out for the association between sex and categorical SDS and SAS values, and education levels and categorical SDS and SAS values.

##### Determination of the associations of depression and anxiety with ECG measurements

Associations between anxiety and depressive symptoms, respectively, and ECG outcomes, and between anxiety and depressive symptoms collectively, and ECG outcomes were determined. Participants were categorized into several groups according to the severity of depression and anxiety symptoms. Associations of continuous SDS and SAS scores with ECG indices were examined using Pearson product-moment correlation coefficients and multivariate regression analysis. One-way ANOVA was performed to estimate the associations of anxiety only, depression only, comorbid anxiety and depression, and no symptom/low severity of both anxiety and depression with ECG measurements before and after adjusting for demographics (sex, age, and education level). Covariates included sociodemographic characteristics. Analyses of covariates were performed to adjust for potentially confounding factors (i.e., demographic characteristics including age, sex, and education level).

##### Prediction analysis

Logistic regression analysis was used to predict the severity of depression and anxiety based on the collected ECG indices.

## Results

### Participants' characteristics

A total of 61 outpatients were included. Of these participants, 9 (14.75%) did not provide SDS and SAS scores. Given the small sample size, we retained the missing reports and replaced them with the mean values. Descriptive analyses of the participants' demographic and clinical characteristics are presented in Table 1. Among the 61 outpatients (mean [standard deviation, SD] age: 37.84 [13.82] years; 41[67.2%] were female), there were 2 (3.3%) outpatients without depression symptoms, 16 (26.2%) with mild depression, 19 (31.1%) with moderate depression, and 24 (39.3%) with severe depression. Ten (16.4%) outpatients did not have anxiety symptoms, 19 (31.1%) exhibited mild anxiety, 20 (32.8%) exhibited moderate anxiety, and 12 (19.7%) exhibited severe anxiety. Only 1 (1.6%) outpatient exhibited neither depression nor anxiety, 9 (14.8%) and 1 (1.6%) outpatients only exhibited depression and anxiety, respectively, and most outpatients (50 [82.0%]) had comorbid depression and anxiety symptoms. Table 1 also displays the ECG measurements, including heart rate (73.16 ± 11.06 BPM), PR interval (153.12 ± 19.13 ms), QT interval (375.67 ± 24.80 ms), QTC (415.95 ± 22.52 ms), and QRS complex (92.13 ± 11.07 ms), of the participants. The interrelationships between ECG indices for these participants were as follows: heart rate and QT interval were significantly negatively correlated (r = −0.547, *p* < 0.01); QTC and heart rate were significantly positively correlated (r = 0.321, *p* = 0.012 <0.05); QT interval and QTC were significantly positively correlated (r = 0.489, *p* < 0.01).

**Table 1:** Demographic and clinical characteristics of outpatients.

	**Total**	**Depression group**					**Anxiety group**					**Symptom group**					**Comorbid group**	
**Characteristic**		**SDS-1**	**SDS-2**	**SDS-3**	**SDS-4**		**SAS-1**	**SAS-2**	**SAS-3**	**SAS-4**		**NONE**	**depression-only**	**anxiety-only**	**co-occurrence**		**No co-occurrence**	
	**(*****n*** **= 61)**	**(*****n*** **= 2)**	**(*****n*** **= 16)**	**(*****n*** **= 19)**	**(*****n*** **= 24)**	***p-value***	**(*****n*** **= 10)**	**(*****n*** **= 19)**	**(*****n*** **= 20)**	**(*****n*** **= 12)**	***p-value***	**(*****n*** **= 1)**	**(*****n*** **= 9)**	**(*****n*** **= 1)**	**(*****n*** **= 50)**	***p-value***	**(*****n*** **= 11)**	***p*** -value^*^
	**mean ± sd;** **n (%)**	**mean ± sd;** ***n*** **(%)**	**mean ± sd;** ***n*** **(%)**	**mean ± sd;** ***n*** **(%)**	**mean ± sd;** ***n*** **(%)**		**mean ± sd;** ***n*** **(%)**	**mean ± sd;** ***n*** **(%)**	**mean ± sd;** ***n*** **(%)**	**mean ± sd;** ***n*** **(%)**		**mean ± sd;** ***n*** **(%)**	**mean ± sd;** ***n*** **(%)**	**mean ± sd;** ***n*** **(%)**	**mean ± sd;** ***n*** **(%)**		**mean ± sd;** ***n*** **(%)**	
**Demographics**																		
Age (years)	37.8 ± 13.8	45.5 ± 10.6	43.8 ± 14.3	38.6 ± 16.5	32.6 ± 9.4	0.065	36.1 ± 12.4	41.0 ± 15.1	41.3 ± 13.4	28.5 ± 9.8	0.044	38	35.9 ± 13.1	53	37.9 ± 14.1	0.719	37.6 ± 12.8	0.958
Sex (Female, n (%))	41 (67.2%)	2 (100%)	8 (50%)	11 (57.9%)	20 (83.3%)	0.082	6 (60%)	14 (73.7%)	13 (65.0%)	8 (66.7%)	0.886	1 (100%)	5 (55.6%)	1 (100%)	34 (68.0%)	0.672	7 (63.6%)	0.78
**Education attainment**																		
% Primary school educated	2 (3.3%)	0	1 (6.3%)	1 (5.3%)	0	0.762	0	0	1 (5.0%)	1 (8.3%)	0.682	0	0	0	2 (4.0%)	0.638	0	0.499
% Junior high school or equivalent educated	14 (23.0%)	0	4 (25%)	7 (36.8%)	3 (12.5%)		3 (30%)	3 (15.8%)	5 (25.0%)	3 (25.0%)		0	3 (33.3%)	0	11 (22.0%)		3 (27.3%)	
% High school or equivalent educated	17 (27.9%)	1 (50%)	4 (25%)	4 (21.1%)	8 (33.3%)		1 (10%)	6 (31.6%)	6 (30.0%)	4 (33.3%)		0	1 (11.1%)	1 (100%)	15 (30.0%)		2 (18.2%)	
% Junior college educated	8 (13.1%)	0	3 (18.8%)	2 (10.5%)	3 (12.5%)		2 (20%)	3 (15.8%)	3 (15.0%)	0		0	2 (22.2%)	0	6 (12.0%)		2 (18.2%)	
% Undergraduate educated	16 (26.2%)	1 (50%)	2 (12.5%)	5 (26.3%)	8 (33.3%)		2 (20%)	5 (26.3%)	5 (25.0%)	4 (33.3%)		1 (100%)	1 (11.1%)	0	14 (28.0%)		2 (18.2%)	
% Graduate educated	4 (6.6%)	0	2 (12.5%)	0	2 (8.3%)		2 (20%)	2 (10.5%)	0	0		0	2 (22.2%)	0	2 (4.0%)		2 (18.2%)	
**ECG measures**																		
Heart rate (bpm)	73.2 ± 11.1	70.5 ± 3.5	77.1 ± 12.9	73.3 ± 8.5	70.7 ± 11.7	0.356	80.0 ± 15.0	69.2 ± 9.9	72.9 ± 10.1	74.2 ± 8.7	0.092	68	81.3 ± 15.3	73	71.8 ± 9.8	0.114	79.4 ± 14.4	0.039
P-R interval (ms)	153.1 ± 19.1	154.5 ± 10.6	151.3 ± 16.6	155.3 ± 21.2	152.5 ± 20.3	0.937	152.4 ± 19.9	154.8 ± 17.7	153.5 ± 22.8	150.4 ± 15.8	0.941	162	151.3 ± 20.7	147	153.4 ± 19.4	0.942	151.9 ± 18.9	0.82
QT interval (ms)	375.7 ± 24.8	365.5 ± 2.1	366.1 ± 29.9	381.4 ± 23.0	378.4 ± 22.3	0.26	354.6 ± 22.4	382.9 ± 26.6	379.1 ± 21.3	376.1 ± 22.1	0.022	367	353.2 ± 23.3	364	380.1 ± 23.4	0.021	355.5 ± 21.4	0.002
QTC (ms)	416.0 ± 22.5	397.0 ± 8.5	412.6 ± 25.2	420.9 ± 25.2	415.8 ± 18.7	0.452	406.7 ± 19.9	417.1 ± 23.9	418.4 ± 26.0	417.8 ± 15.5	0.572	391	408.4 ± 20.3	403	418.1 ± 22.9	0.4	406.4 ± 18.9	0.12
QRS complex	92.13 ± 11.1	87.5 ± 9.2	93.2 ± 12.7	91.6 ± 12.9	92.2 ± 8.8	0.915	90.3 ± 13.1	90.5 ± 10.7	94.5 ± 11.5	92.3 ± 9.6	0.665	81	91.3 ± 13.5	94	92.5 ± 10.8	0.779	90.6 ± 12.5	0.625
**Psychological measures**																		
SDS	68.8 ± 11.4	44.5 ± 2.1	56.6 ± 2.5	67.2 ± 2.5	80.3 ± 5.8	0.000	56.4 ± 6.6	65.0 ± 10.9	72.0 ± 5.4	79.9 ± 10.4	0.000	43	57.9 ± 4.9	46	71.7 ± 10.0	0.000	55.5 ± 7.0	0.000
SAS	59.6 ± 12.7	46.0 ± 17.0	49.6 ± 9.1	58.2 ± 9.4	68.6 ± 10.6	0.000	40.1 ± 5.2	55.0 ± 2.8	62.9 ± 3.3	77.9 ± 7.6	0.000	34	40.8 ± 5.0	58	63.6 ± 9.9	0.000	41.7 ± 7.3	0.000

### Correlations between the participants' demographic characteristics and ECG measurements

Correlations between demographic characteristics and ECG measurements were analyzed because previous studies have suggested that sex, age, and education level were related to ECG measurements (25–27). Independent *t*-tests showed that male outpatients had significantly higher QRS complex amplitude than females (*p* = 0.002). No significant differences were found in other ECG indices between genders. Pearson correlation analysis indicated that age was only significantly associated with QTC interval (r = 0.450, *p* < 0.01). No associations were found between education level and ECG indices as revealed by one-way ANOVA.

### Correlations between the participants' demographic characteristics and psychological measurements

Correlations between demographic characteristics and psychological measurements were also analyzed. Age was found to be significantly correlated with SDS scores (r = −0.294, *p* = 0.021), but not with continuous SAS scores (r = −0.173, *p* = 0.182). However, age was significantly related to anxiety severity (*p* = 0.044) but not with depression severity (*p* = 0.065).

Chi-squared tests were used to examine relationships between gender, education level and SDS score, SAS score, and disorder status. There was no significant association between sex and SDS score (*p* = 0.082), SAS score (*p* = 0.882), disease status (*p* = 0.672) and the status of comorbidity (*p* = 0.780). The associations between education level and SDS score (*p* = 0.762), SAS score (*p* = 0.682), disease status (*p* = 0.638), and the status of comorbidity (*p* = 0.499) were also not significant.

### Correlations of depression and anxiety symptoms with ECG measurements

#### Associations between depression and ECG measurements

**Figure 1:**
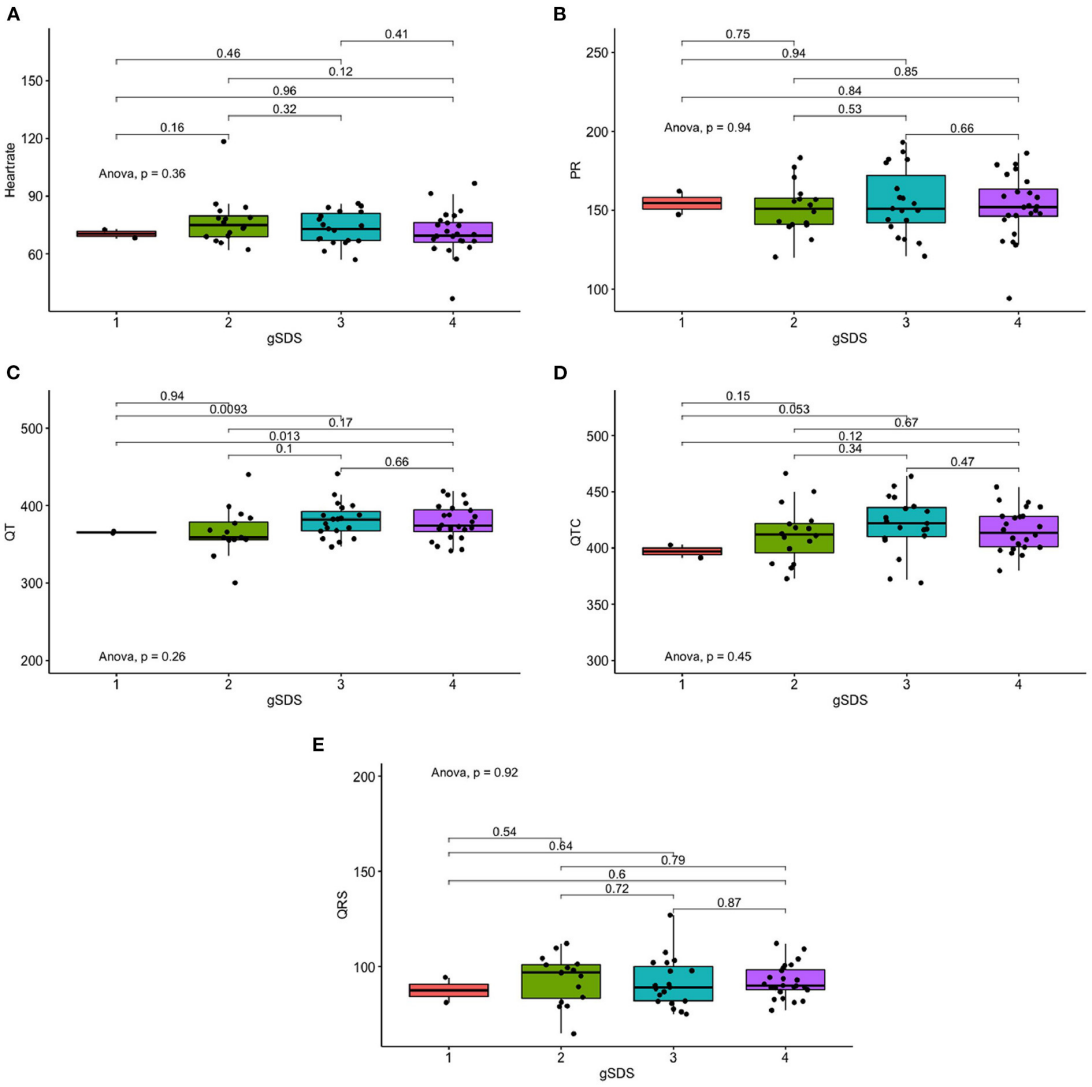
Differences in ECG measures of depression severity. **(A)** Differences in Heart rate of depression severity. **(B)** Differences in PR of depression severity. **(C)** Differences in QT of depression severity. **(D)** Differences in QTC of depression severity. **(E)** Differences in QRS measures of depression severity.

**Figure 2:**
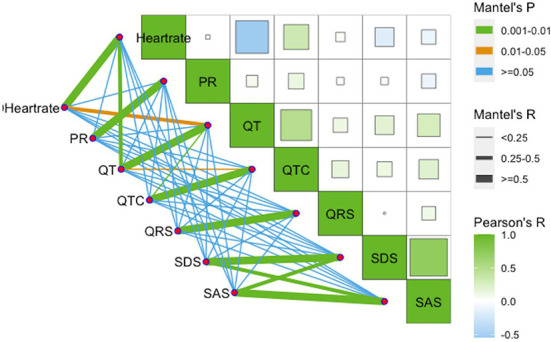
Correlation between ECG measures and psychological variables.

We conducted a one-way ANOVA on depression status and ECG indices (Table 2, Figure 1), and calculated Pearson correlation coefficients for continuous SDS scores and ECG indices (Figure 2). Correlations between depression status and all five ECG indices were not significant. After adjusting for age, sex, and education level, the correlations remained insignificant (Table 3).

**Table 2:** Differences in ECG measures of depression and anxiety severity.

**ECG measures**	**SDS**			**SAS**		
	**F**	**p**	**η^2^**	**F**	**p**	**η^2^**
**Heart rate (bpm)**	1.102	0.356	0.055	2.253	0.092	0.106
**P-R interval (ms)**	0.138	0.937	0.007	0.132	0.941	0.007
**QT interval (ms)**	1.373	0.260	0.067	3.454	0.022	0.154
**QTC (ms)**	0.889	0.452	0.045	0.673	0.572	0.034
**QRS complex**	0.171	0.915	0.009	0.527	0.665	0.027

**Table 3:** Association between depression severity and ECG measures.

**ECG measures**	**Unadjusted mean**					**Adjusted mean** ^*^				
	**SDS-1**	**SDS-2**	**SDS-3**	**SDS-4**	**P^a^**	**SDS-1**	**SDS-2**	**SDS-3**	**SDS-4**	P^*^
	**mean ±sem**	**mean ±sem**	**mean ±sem**	**mean ±sem**		**mean ±sem**	**mean ±sem**	**mean ±sem**	**mean ±sem**	
**Heart rate (bpm)**	70.5 ± 2.5	77.063 ± 3.215	73.263 ± 1.948	70.708 ± 2.386	0.356	70.840 ± 8.128	76.593 ± 2.936	72.826 ± 2.643	71.339 ± 2.439	0.598
**P-R interval (ms)**	154.5 ± 7.5	151.313 ± 4.139	155.316 ± 4.858	152.458 ± 4.148	0.937	153.178 ± 13.884	148.117 ± 5.016	154.669 ± 4.514	155.211 ± 4.166	0.725
**QT interval (ms)**	365.5 ± 1.5	366.063 ± 7.484	381.421 ± 5.287	378.375 ± 4.547	0.26	358.495 ± 17.092	363.538 ± 6.175	381.625 ± 5.557	380.480 ± 5.129	0.092
**QTC (ms)**	397 ± 6.0	412.625 ± 6.297	420.895 ± 5.774	415.833 ± 3.822	0.452	388.105 ± 13.932	408.800 ± 5.033	420.570 ± 4.53	419.382 ± 4.181	0.066
**QRS complex**	87.5 ± 6.5	93.188 ± 3.17	91.632 ± 2.965	92.208 ± 1.797	0.915	89.189 ± 7.429	90.320 ± 2.684	90.519 ± 2.415	94.860 ± 2.229	0.507

* adjusted for age, sex, and education levels.

a unadjusted p values were calculated.

#### Associations between anxiety and ECG measurements

**Figure 3:**
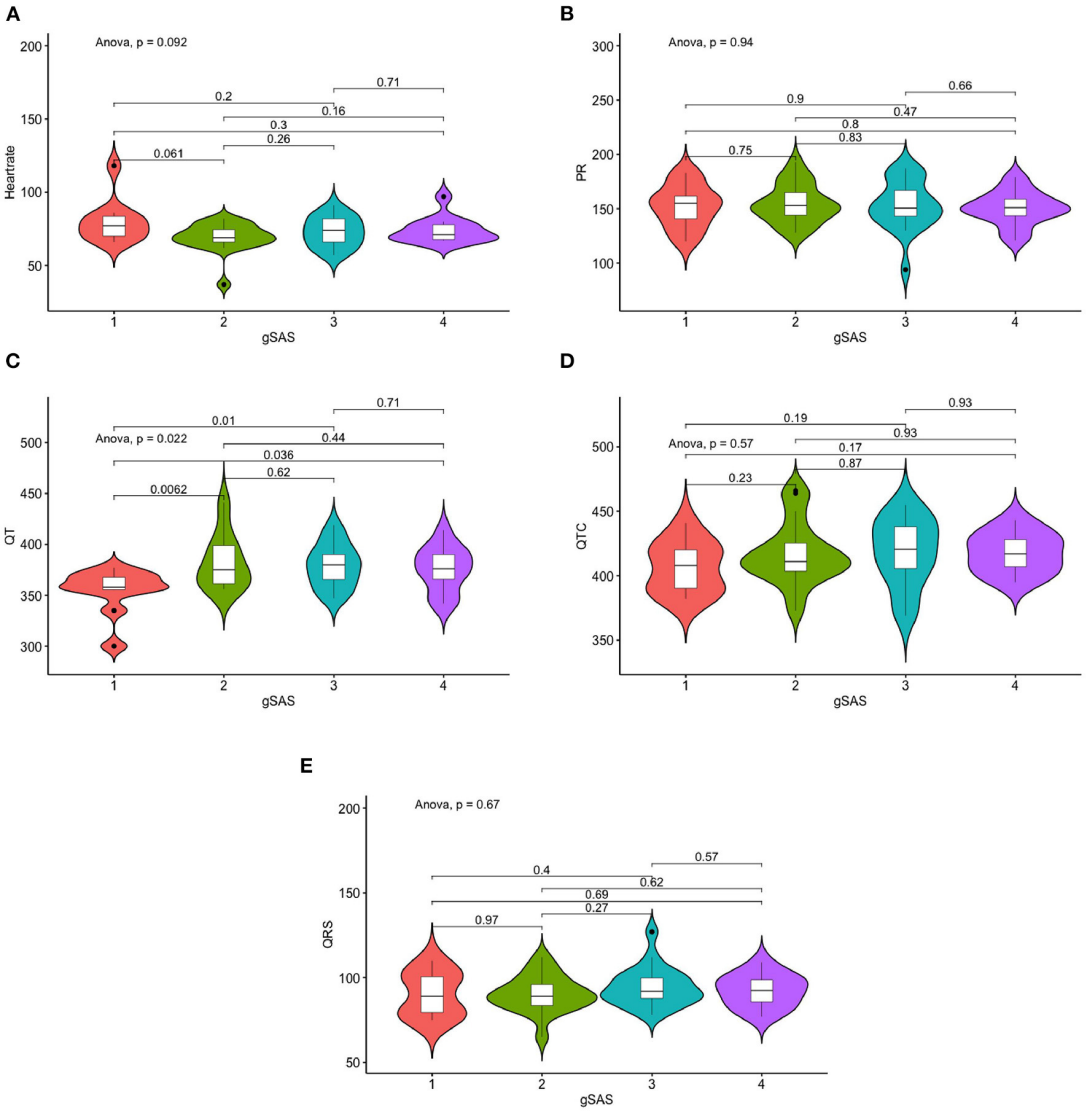
Differences in ECG measures of anxiety severity. **(A)** Differences in Heart rate of anxiety severity. **(B)** Differences in PR of depression severity. **(C)** Differences in QT of depression severity. **(D)** Differences in QTC of depression severity. **(E)** Differences in QRS measures of depression severity.

Anxiety severity significantly differs in QT interval (*p* = 0.022), but not with other ECG indices (Table 2, Figure 3). Similarly, continuous SAS scores were significantly correlated with QT interval (r = 0.263, *p* = 0.04), but not with other ECG indices (Figure 1). Adjusting for age, sex, and education level, attenuated the significance of this correlation (adjusted *p* = 0.041) (Table 4).

**Table 4:** Association between anxiety severity and ECG measures.

**ECG measures**	**Unadjusted mean**					**Adjusted mean** ^*^				
	**SDS-1**	**SDS-2**	**SAS-3**	**SAS-4**	**P^a^**	**SAS-1**	**SAS-2**	**SAS-3**	**SAS-4**	P^*^
	**mean ±sem**	**mean ±sem**	**mean ±sem**	**mean ±sem**		**mean ±sem**	**mean ±sem**	**mean ±sem**	**mean ±sem**	
**Heart rate (bpm)**	80 ± 4.749	69.211 ± 2.26	72.9 ± 2.259	74.167 ± 2.525	0.092	80.424 ± 3.412	68.997 ± 2.539	72.193 ± 2.427	75.331 ± 3.344	0.066
**P-R interval (ms)**	152.4 ± 6.277	154.842 ± 4.067	153.45 ± 5.096	150.417 ± 4.547	0.941	152.549 ± 6.188	153.999 ± 4.605	152.293 ± 4.402	153.556 ± 6.065	0.993
**QT interval (ms)**	354.6 ± 7.071	382.947 ± 6.109	379.05 ± 4.754	376.083 ± 6.381	0.022	355.793 ± 7.408	381.149 ± 5.513	377.762 ± 5.27	380.083 ± 7.261	0.041
**QTC (ms)**	406.7 ± 6.288	417.105 ± 5.491	418.4 ± 5.822	417.75 ± 4.462	0.572	409.087 ± 6.353	414.102 ± 4.729	415.466 ± 4.52	425.406 ± 6.227	0.325
**QRS complex**	90.3 ± 4.15	90.474 ± 2.463	94.5 ± 2.576	92.333 ± 2.762	0.665	89.885 ± 3.287	90.268 ± 2.447	93.772 ± 2.339	94.219 ± 3.222	0.596

* adjusted for age, sex, and education levels.

a unadjusted p-values were calculated.

#### Associations between comorbid depression and anxiety and ECG measurements

Depression and anxiety levels were significantly positively correlated (r = 0.717, *p* < 0.01). When examining SDS and SAS scores simultaneously as continuous variables using multivariate regression analyses, it was found that SDS score was most strongly correlated with heart rate (β = −0.215, *p* = 0.249), while SAS score was most strongly correlated with QT interval (β = 0.275, *p* = 0.136). In addition, the comorbidity of anxiety and depression was significantly correlated with heart rate (*p* = 0.039) and QT interval (*p* = 0.002). Furthermore, disorder status only significantly differed with different QT intervals (*p* = 0.021).

### Prediction of symptom status using ECG measurements

We tried to predict symptom status using the five ECG indices. Four symptom statuses were defined in this study: (1) depression severity: 1-normal, 2-mild, 3-moderate, 4-severe; (2) anxiety severity: 1-normal, 2-mild, 3-moderate, 4-severe; (3) comorbid severity: 1-no symptom/low severity of both anxiety and depression, 2-depression only, 3-anxiety only, 4-comorbid anxiety and depression; (4) status of comorbidity: 1-none comorbid, 2-comorbid depression and anxiety.

To predict the depression severity, anxiety severity and comorbid severity, we conducted logistic regression analyses. However, we could not predict these three symptom statuses based on the available ECG measurements.

To predict the status of comorbidity, we used logistic regression analyses (binary logistic regression; forward logistic regression). Results suggested that QT interval was the only significant predictor for the status of comorbidity (*p* = 0.01, β = 0.058, odds ratio [OR] = 1.059).

## Discussion

Although a moderate to strong relationship between depression and anxiety symptoms and ECG features has been reported in previous literature, contradicting results generated from these studies are frustrating. Additionally, little has been done on predicting the severity of depression and anxiety. To our knowledge, this study is the first attempt to predict the severity of depression, anxiety, and their status of comorbidity using ECG indices.

Findings from the present study showed that continuous and categorical anxiety scores were significantly correlated with QT interval. These findings are consistent with those of Lapidus et al. (28), who discovered that a high level of anxiety was associated with increased QT dispersion, which may predispose to cardiac arrhythmias (28). Surprisingly, the current study did not detect any evidence of a significant correlation between depression and ECG indices. However, because of the small sample size of our study, caution must be taken in interpreting our observations. Prior research also yielded heterogeneous findings (significant or non-significant) on the relationship between depression symptoms and ECG measurements (29, 30).

The simultaneous analyses of continuous SDS and SAS scores revealed significant associations of depression with heart rate, and anxiety and QT interval. Therefore, it seems reasonable to determine whether the comorbidity of anxiety and depression is significantly correlated with heart rate and QT interval. Additionally, disease status only differed significantly with different QT intervals. Noteworthy, we could not detect a statistically significant relationship between depression and ECG indices until taking anxiety into account. This agrees with prior research showing that anxiety, but not depression, negatively influenced parasympathetic modulation of heart rate, suggesting that anxiety may be more related to adverse cardiological outcomes (15). However, some research also suggests that the association between anxiety and heart disease may be responsible for the comorbidity of depression (31). This inconsistency in causality may be due to the specific ECG indices selected, as prior research also found that benign palpitation was significantly associated with anxiety, but not depression (32). Another factor to consider is the stage of disease, as aforementioned in the introduction. For example, one study has suggested that anxiety may play different roles in different stages of depression in individuals with inherited cardiac disorders (33).

Another important finding of our study is that QT interval was the only ECG index that can be used to predict the comorbidity of depression and anxiety. Likewise, the reliability of using ECG features obtained from wearable devices for diagnosing anxiety has been validated (34), and a dose-response relationship has been found between the severity of depression and the risk of coronary heart disease (35). In addition, a prior study used heart rate variability to effectively discriminate between depression and anxiety patients (10). Another study also predicted depressed patients with suicidal ideation based on ECG recordings (36). In summary, it is possible to predict the severity of depression and anxiety using ECG indices.

## Limitations and future work

The present study has many limitations to acknowledge. First, despite the robust risk adjustment during statistical calculation, confounding effects as a result of unmeasured variables, such as baseline health status, lifestyle (e.g., diet habits, exercise habits), and current medication status, cannot be excluded. Future studies will be needed to further investigate these variables. In addition, it will be important for future studies to investigate how the other remaining ECG indices correlate with depression and anxiety symptoms. Second, there was a potential selection bias considering the small sample size of our study. Therefore, findings obtained based on this small sample may not be generalizable to other settings. However, as we aimed to investigate the association between ECG indices and earlier depression and anxiety symptoms in outpatients with first-episode depression, the sample used in this study was relatively representative. In the future, it is important to verify our results in longitudinal analyses with repeated measures in a large, high-risk population. Third, we only used SDS and SAS as depression and anxiety assessment tools, respectively. Due to the subjective nature of these two scales, recall bias may exist, and the evidence may be insufficient for establishing diagnoses. We believe that the correlation and prediction power would have been more statistically significant if more strict diagnoses were established. Fourth, although associations between ECG indices and depression and anxiety have been identified in this study, the causal mechanisms remain to be elucidated. Additionally, prospective studies are needed to clarify the pharmacological roles of depression and anxiety in the management of heart disease.

## Conclusion

In conclusion, the present study demonstrated that QT interval was most strongly associated with and was the only significant predictor for the comorbidity of depression and anxiety. These findings have important implications for the prevention and intervention of depression and anxiety and highlight the need to consider psychological factors and established predictors when assessing a person's risk of heart disease. We believe that these data will have reference value for health care providers and hospital administrators.

## Data availability statement

The raw data supporting the conclusions of this article will be made available by the authors, without undue reservation.

## Ethics statement

The studies involving human participants were reviewed and approved by the Shanghai Pudong New Area Mental Health Ethics Committee. The patients/participants provided their written informed consent to participate in this study.

## Author contributions

Full access to all of the data in the study and take responsibility for the integrity of the data, accuracy of the data analysis, obtained funding, administrative, technical, material support, and supervision: JX and XF. Concept, design, acquisition, analysis, interpretation of data, and critical revision of the manuscript for important intellectual content: JX, XF, and MT. Drafting of the manuscript and statistical analysis: MT. All authors contributed to the article and approved the submitted version.

## Funding

This study was supported by the Research Project of Shanghai Science and Technology Commission (20dz2260300) and The Fundamental Research Funds for the Central Universities; Science and Technology Development Fund of Shanghai Pudong New Area, PKJ2020-Y34; Medical discipline; China Key Project of Science and Technology Innovation 2030 (Grant No. 2021ZD0200535).

## Conflict of interest

The authors declare that the research was conducted in the absence of any commercial or financial relationships that could be construed as a potential conflict of interest.

## Publisher's note

All claims expressed in this article are solely those of the authors and do not necessarily represent those of their affiliated organizations, or those of the publisher, the editors and the reviewers. Any product that may be evaluated in this article, or claim that may be made by its manufacturer, is not guaranteed or endorsed by the publisher.
